# BMP Signaling in the Development and Regeneration of Cranium Bones and Maintenance of Calvarial Stem Cells

**DOI:** 10.3389/fcell.2020.00135

**Published:** 2020-03-10

**Authors:** Guiqian Chen, Haodong Xu, Yifeng Yao, Tingting Xu, Mengting Yuan, Xingen Zhang, Zhengbing Lv, Mengrui Wu

**Affiliations:** ^1^Zhejiang Provincial Key Laboratory of Silkworm Bioreactor and Biomedicine, College of Life Sciences and Medicine, Zhejiang Sci-Tech University, Hangzhou, China; ^2^Department of Orthopedics, Zhejiang Rongjun Hospital, Jiaxing, China; ^3^Institute of Genetics, Life Science College, Zhejiang University, Hangzhou, China

**Keywords:** BMP signaling, neural-crest cells, suture stem cell, calvarial regeneration, mesoderm

## Abstract

The bone morphogenetic protein (BMP) signaling pathway is highly conserved across many species, and its importance for the patterning of the skeletal system has been demonstrated. A disrupted BMP signaling pathway results in severe skeletal defects. Murine calvaria has been identified to have dual-tissue lineages, namely, the cranial neural-crest cells and the paraxial mesoderm. Modulations of the BMP signaling pathway have been demonstrated to be significant in determining calvarial osteogenic potentials and ossification *in vitro* and *in vivo*. More importantly, the BMP signaling pathway plays a role in the maintenance of the homeostasis of the calvarial stem cells, indicating a potential clinic significance in calvarial bone and in expediting regeneration. Following the inherent evidence of BMP signaling in craniofacial biology, we summarize recent discoveries relating to BMP signaling in the development of calvarial structures, functions of the suture stem cells and their niche and regeneration. This review will not only provide a better understanding of BMP signaling in cranial biology, but also exhibit the molecular targets of BMP signaling that possess clinical potential for tissue regeneration.

## Introduction

The murine calvaria is evolutionally patterned and well developed. The calvaria provides important protection for the growth and formation of the brain, and its growth is concordantly orchestrated across developmental stages. Since the establishment of the dual-tissue lineages of the calvaria (Jiang et al., 2002), studies are focusing on region-dependent differential regulation of calvarial bone development (Quarto et al., 2009, 2010, 2018; Wiren et al., 2011; Ichikawa et al., 2015; Li et al., 2015; Doro et al., 2019). On one hand, the region-dependent roles of evolutionally conserved signaling pathways in calvarial bones are under clarifying (Quarto et al., 2009; Behr et al., 2010; Li et al., 2010; Li et al., 2015). On the other hand, high-throughput sequencing results indicate that gene-regulatory networks are differently enriched in different segments of calvarial bones (Homayounfar et al., 2015; Hu et al., 2017; Wu et al., 2017; Chen et al., 2019a), both of which have the potential to explain the regional differences upon osteogenic capacities, ossification, and regeneration in the calvarial bones.

The bone morphogenetic protein (BMP) signaling pathway has been demonstrated to be an important regulator in the shaping of the skeletal system, patterning the neural crest and craniofacial development (Mishina and Snider, 2014; Graf et al., 2016). This pathway is transduced through the binding of BMP ligands to BMP receptor (BMPR) type I and type II (BMPRI and BMPRII), which further activate the intracellular Smads (Smad1, Smad5, and Smad8) proteins, and Smads phosphorylation can associated with co-Smad4 into a complex, which can translocate into the nucleus and trigger bone-related gene expression. Deficiencies of the BMP signaling pathway at different cellular levels lead to a malformation of the skeleton and birth defects in a tissue-specific manner (Nie et al., 2006; Trainor, 2010; Chen et al., 2012; Bhatt et al., 2013; Graf et al., 2016). Here, the versatile regulatory functions of the BMP signaling pathway in orchestrating the homeostasis of the stem-cell niche and the calvarial bones with dual-tissue lineages are summarized.

## BMP Signaling in Tissue-Derived Osteoblasts

### The Calvarial Bones Have Two Tissue-Lineages

Using genetic mouse model, the murine calvaria has been demonstrated originated from with dual-tissue lineages (Jiang et al., 2002; Kuratani, 2005), namely, the cranial neural-crest cells (CNC) and paraxial mesoderm mesenchymal stem cells. The CNC cells that originate from the dorsal neural tube appear early during embryogenesis, and can diversify into multiple cell types, and contribute to most cranial bones, including the nasal-frontal bones, maxillary, frontal bone, and mandible (Chai and Maxson, 2006). Paraxial mesoderm-derived cells contribute to the formation of parietal bone (Jiang et al., 2002; Kuratani, 2005). Both CNC-derived and paraxial mesoderm derived osteoprogenitor cells undergo intramembranous ossification to produce cranial bones. Some bones in the cranial base are also from CNC, but they are formed via endochondral ossification, where mesenchymal cells first differentiate into the chondrocytes to form the cartilage primordial. The intramembranous ossification happens with a direct differentiation into osteoblasts progenitors from the mesenchymal cells (Mishina and Snider, 2014). Different bones are connected by different sutures. Nasal and metopic sutures are derived from CNC, and coronal sutures are derived from mesoderm, which connect CNC-derived frontal bone and mesoderm-derived parietal bone, and the sagittal suture is derived from CNC, which separate two mesoderm-derived parietal bone (Mishina and Snider, 2014). However, CNC-derived and paraxial mesoderm derived osteoblasts show distinct differences in osteogenic potential, the regenerative capacities and ossification (Reichert et al., 2013).

**FIGURE 1:**
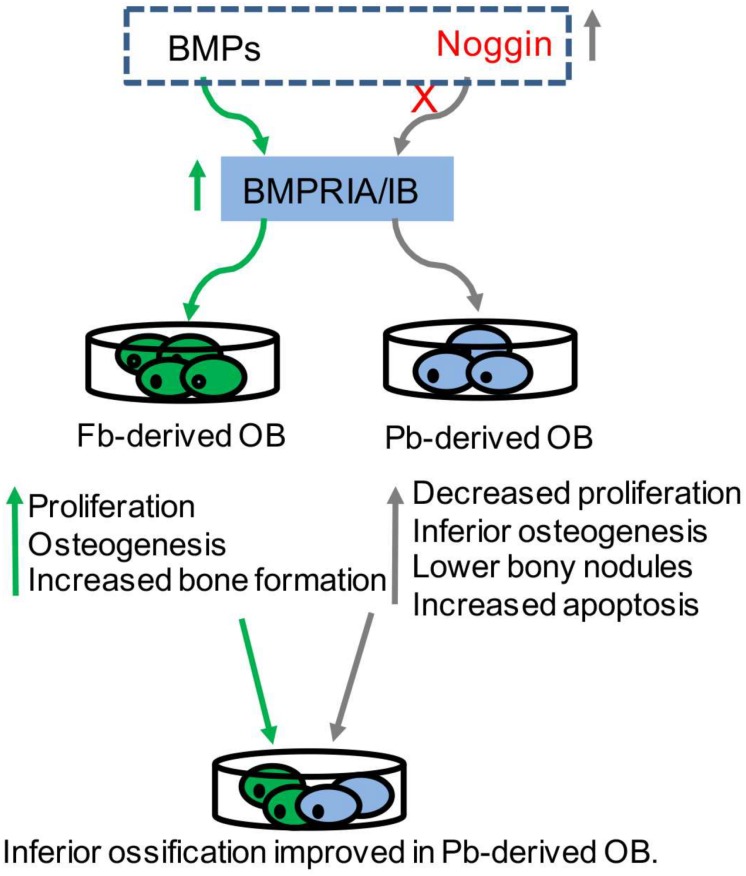
BMP signaling in tissue-derived osteoblasts. BMPRs (BMPRIA/IB) were highly expressed in neural-crest-derived frontal osteoblasts (Fb-derived OB) (green in arrow), which exhibited increased proliferation, and osteogenesis and bone formation. Noggin was highly expressed in mesoderm-derived parietal osteoblasts (Pb-derived OB), which exhibited decreased proliferation, inferior osteogenesis, lower bone formation and increased apoptosis (gray in arrow). The addition of some Fb-derived OB into Pb-derived OB can significantly improve the ossification. Proper modulation of BMP signaling (dotted box) can influence the osteogenic potential in tissue-derived osteoblasts.

The main difference between CNC-derived osteoblasts and mesoderm-derived osteoblasts has been demonstrated *in vitro* (Xu et al., 2007), namely, CNC-derived osteoblasts display robust proliferation, and the extent of the cell differentiation is much less, and the extent of bone formation is faster compared to mesoderm-derived osteoblasts, exhibiting minimal capacities of bone nodules formation *in vitro* (Xu et al., 2007). When mesoderm-derived osteoblasts are cultured with the addition of CNC-derived osteoblasts, the inferior performance of ossification in mesoderm-derived osteoblasts have been improved (Doro et al., 2019), suggesting that CNC input can favor the osteogenic capacities and the extent of ossification (Doro et al., 2019) (Figure 1).

### The Levels of BMP Signaling in Tissue-Derived Osteoblasts

Bone morphogenetic protein signaling in bone has been reviewed previously (Nie et al., 2006; Chen et al., 2012; Graf et al., 2016; Wu et al., 2016). Briefly, BMP ligands bind to their receptors in the membrane, triggering phosphorylation of R-Smads (Smad1, Smad5, and Smad9) that complex with co-Smad (Smad4) and translocate into the nucleus to drive target gene expressions. BMP-Smad signaling is well-known to be regulated by extracellular antagonists (e.g., Noggin) and intracellular inhibitors (e.g., Smad6 and Smad7). In a previous study, BMPRs were found with higher expressions in CNC-derived osteoblasts, while the expressions of the Noggin were higher in mesoderm-derived osteoblasts compared to that in CNC-derived osteoblasts from 2 to 5-day-old mice (Xu et al., 2007). Based on our high-through sequencing data, the level of BMPRs in embryonic frontal bone tissues were higher than that in embryonic parietal bone tissues (Hu et al., 2017). The inhibition of BMP signaling using Noggin results in increased apoptosis and osteogenesis in CNC-derived osteoblasts, and similarly, the exogenous stimulation of BMP signaling using BMP2 results in reduced apoptosis and osteogenesis in mesoderm-derived osteoblasts (Senarath-Yapa et al., 2013), suggesting that the modulation of BMP signaling *in vitro* is able to influence the extent of osteogenic potentials in CNC- and mesoderm-derived osteoblasts (Figure 1).

### Functions of BMP Signaling in the Development of Cranial Bones

There are 15 BMPs in humans and rodents. Among them, BMP2, BMP4, and BMP7, as well as growth differentiation factor 5 (GDF5) are essential for embryonic skeletal development, while BMP6, BMP7, and GDF6 are essential for late stages of skeletal development (Graf et al., 2016; Wu et al., 2016). A number of BMPs are expressing in craniofacial bones in a temporospatial manner, including BMP2, BMP4, BMP3, BMP5, BMP6, and BMP7 as well as GDF1 and GDF6. Genetic mouse models have been used to verify the functions of BMP signaling in calvarial bones *in vivo*. In CNC cells, the deletion of BMP2 using *Wnt1-Cre* leads to craniofacial anomalies that resemble the symptoms of the Pierre Robin sequence (PRS), including smaller craniofacial bones (Chen et al., 2019c). Mutation of BMP2 in CNC leads to abnormal coordination between the proliferation and differentiation of osteogenic progenitors (Chen et al., 2019c). GDF6 is expressed in the primordia of mouse frontal bones, and GDF6 removal results in coronal suture fusion and defective frontal and parietal bones. The accelerated differentiation of suture mesenchyme was found earlier than the onset of calvarial ossification (Clendenning and Mortlock, 2012). BMP4 is a major regulator in shaping the craniofacial cartilage (Albertson et al., 2005). Interestingly, the inactivation of BMP2 and BMP4 using *Wnt1-Cre* in preosteoblasts and periosteal dura can result in defective skull and cerebral veins. BMP2/BMP4, which can be secreted from CNC or mesoderm-derived preosteoblasts and dura, can function in a paracrine manner to regulate the morphogenesis of the cerebral veins (Tischfield et al., 2017), revealing the unrecognized importance of BMP signaling in the maintenance of tissue–tissue interactions for craniofacial organ growth (Table 1).

**TABLE 1:** Functions of BMP signaling in the development of cranial bones.

Gene	Model	Defects	References
BMP2	*Wnt1-Cre*	Smaller craniofacial bones	Chen et al., 2019c
BMP2/BMP4	*Wnt1-Cre*	Defective skull and dural cerebral veins	Tischfield et al., 2017
BMPRIA	*Wnt1-Cre*	Defective temporomandibular joint	Gu et al., 2014
enhanced BMPRIA	*Wnt1-Cre*	Inhibitory osteogenesis	Gu et al., 2014
BMP7	*Wnt1-Cre*	Alteration of oral cavity morphology	Kouskoura et al., 2013
BMP7/BMP4	*Mef2c-Cre*	Defective mesenchymal transition	Bai et al., 2013
BMPRIA	*P0-Cre*	Wide-open anterior fontanelles	Saito et al., 2012
BMPRIA	*Pax3-Cre*	Reduction in neural-crest cells	Stottmann and Klingensmith, 2011
BMPRIA	*Wnt1-Cre*	Post-migratory development of a subset of NCC derivative cell types	Stottmann and Klingensmith, 2011
Smad1	*Col2a1-Cre*	Defective calvarial bone	Wang et al., 2011
Smad4	*Wnt1-Cre*	Defective mid-gestation	Nie et al., 2008
Smad4	*Wnt1-Cre*	Underdevelopment of branchial arch	Ko et al., 2007
ALK2	*Wnt1-Cre*	Impaired neural-crest cells	Kaartinen et al., 2004
ALK2	*Wnt1-Cre*	Multiple craniofacial defects	Dudas et al., 2004
BMPRII	*Prx1-Cre*	Normal skeletons	Gamer et al., 2011
BMPRIA	*Dermo1-Cre*	Defective ventral body wall formation	Sun et al., 2007

Bone morphogenetic protein receptors are heterodimers complex composed of type I receptors and type II receptors. There are also different type I and type II receptors, which create a complex ligand-receptor interaction network and allows for specific outcomes for the skeleton. Among the three type I receptors, BMPRIA has been best-studied and shown to be indispensable for hindbrain neural tube closure (Stottmann and Klingensmith, 2011). Deletion of BMPRIA in CNC using *P0-Cre* leads to 100% abnormal phenotype with wide-open anterior fontanelles. This phenotype in the craniofacial mesenchyme results in an activated p53 apoptosis pathway and a downregulation of c-Myc and Bcl-XL. Therefore, the optimal BMPRIA-mediated signaling is essential for CNC-derived frontal bone development (Saito et al., 2012). Further exploration of the phenotype of the deletion of BMPRIA in CNC cells using *Wnt1-Cre* results in a defective temporomandibular joint (Gu et al., 2014). The constitutive activation of BMPRIA in CNC cells leads to the craniosynostosis, which happened through the induction of p53-mediated apoptosis in nasal cartilage (Hayano et al., 2015). Three type II receptors (BMPRII, ActRIIA, and ActRIIB) were also important for the signaling transduction. Deficiencies of BMPRII, one of the three type II receptors, result in normal skeleton using *Prx1-Cre*, suggesting that different requirements of BMPRs in transducing the signaling to shape the calvaria development (Gamer et al., 2011) (Table 1). However, the roles of other two type II receptors, ActRIIA and ActRIIB, in craniofacial bones are still unclear.

The intracellular mediator Smad1 is needed for bone development, and deficient Smad1 results in defective calvarial bone (Wang et al., 2011). The inactivation of Smad4 in CNC cells leads to birth death, accordingly, the defective mid-gestation and increased cell death (Nie et al., 2008). Additionally, Smad4 deficiency leads to underdevelopment of the first branchial arch (Ko et al., 2007). Improper mutation from a rare transmitted frameshift in inhibitory Smad6 (p. 152 fs^∗^27) can be inherited from non-syndromic craniosynostosis parents (Timberlake et al., 2018), emphasizing the importance of BMP-Smads signaling in shaping CNC-derived craniofacial development.

The orchestration of the BMP signaling pathway eventually converges at crucial transcriptional factors to regulate the osteogenesis and ossification. For example, Msx2, a bona fide downstream target of BMP signaling, regulates the activities of osteoblast-specific transcriptional factors Runx2 and Osterix (Osx). Mutations of Runx2 (Lee et al., 1997; Mundlos et al., 1997) and Osx (Nakashima et al., 2002) lead to severe defects in bone ossification. Msx2 can label a special population of mesenchymal precursor cells in the cranial vault (Sakagami et al., 2018). Deficient Msx1/2 using *Wnt1-Cre* leads to a larger defect in frontal bone (Roybal et al., 2010). A deficiency of Runx2 in CNC cells results in defective ossification, including the frontal bone, mandible, and nasal bone. Runx2 is required both for mesoderm- and CNC-derived cells to differentiate and ossify. But CNC-derived frontal bone is more dependent on the activity of Runx2 (Shirai et al., 2019). Neural crest-specific inactivation of Osx resulted in a complete absence of intramembranous skeletal bones that derived from the CNC. Besides, the CNC-derived endochondral skeletal bones were also affected (Baek et al., 2013). Taken together, the data suggested that a precise responsiveness to BMP signaling in CNC cells is crucial for the proper morphogenesis of the calvaria, and the BMP signaling can be counted on for the superior osteogenic potential in CNC-derived bones.

## BMP Signaling in Calvarial Suture-Derived Stem Cells

### Identification of Calvarial Suture-Derived Stem Cells

The sutures are the center of an environmental niche that containing stem cells, such as Gli1+ stem cells (Zhao et al., 2015; Park et al., 2016), Axin2+ stem cells (Maruyama et al., 2016; Maruyama, 2019), and Prx1+ stem cells (Wilk et al., 2017), where were identified and demonstrating a strong potential to differentiate into osteoblasts and osteocytes at the postnatal stage. Prx1+ suture-derived stem cells are resident in cranial sutures, and they are not detectable in the skin or the dura mater at an embryonic stage (Seo and Serra, 2009), neither in the postnatal periosteum or the dura mater (Wilk et al., 2017). Prx1+ cells can label both CNC- and mesoderm-derived cells. Prx1+ cells are detectable at the osteogenic fronts but does not co-expressed with Osx in the suture-space mesenchyme, and Prx1-derived cells are not expressed in blood cells (Wilk et al., 2017) (Table 2). Gli1+ cells are more populous than the Prx1+ cells in the suture space. Gli1+ cells are detectable throughout the entire periosteum, dura, and suture mesenchyme at birth (Guo et al., 2018), but this pattern gradually becomes undetectable by 1 month postnatal, and Gli1+ cells are not detectable in fontanelles or osteocytes. Gli1+ cells eventually restricted to cranial sutures, including the fused posterior frontal suture, where they remain throughout adulthood (Zhao et al., 2015; Doro et al., 2017). Gli1+ cells can contribute to osteogenic fronts, periosteum, and dura after tamoxifen treatment (Guo et al., 2018; Table 2). Axin2+ cells only label ∼15% of mesenchymal cells, and are highly restricted to the midline of the suture (Maruyama et al., 2016). Axin2+ cells are detectable in osteogenic fronts and the periosteum at birth, and they are almost undetectable at postnatal 1 month (Yu et al., 2005). Axin2+ cells reside in all cranial sutures except the fused posterior frontal suture (Maruyama et al., 2016), and they exhibit a narrower range than the Gli1+ and Prx1+ suture stem cells (Table 2).

**TABLE 2:** Distribution of identified populations of suture-derived stem cells.

Location	Prx1+	Gli1+ (entire suture space)	Axin2+ (≈15%)
Periosteum	−	+ (born) →− (1 m)	+ →− (1 m)
Dura mater	−	+ (born) →− (1 m)	+ →− (1 m)
Patent posterior frontal suture	+	+	+
Fused posterior frontal suture	+	−	−
Coronal suture	++	+++	+
Sagittal suture	++	+++	+
Lambdoid suture	++	+++	+
Osteocytes	−	−	−
Osteogenic fronts	+	−	+
Blood cells	−	−	−

### The Potential BMP Mediated Contribution of CNC to Calvarial Stem Cells

Prx1+, Axin2+, and Gli1+ suture-derived stem cells account for most cranial sutures, and most cranial sutures come from CNC cells, such as the sagittal suture and posterior frontal suture, and the coronal suture is a mixture of CNC- and mesoderm-derived cells. CNC cells are multi-potent cells, although they are transient during the development. It is still not clear that how suture-derived stem cells have been generated or maintained, and one possibility is that CNC migrated there and some cells keep the stem-cell-like feature in the sutures. The other possibility is that some genes were specially expressed to maintain stem-cell feature. But no evidences of early expressions of Prx1, Gli, or Axin2 at E8.5 when the CNC induced, some are expressed in post-migratory neural-crest cells, such as Prx1 is particularly expressed in post-migratory neural-crest cells (Thomas et al., 1998; Ishii et al., 2012), and Axin2 is highly activated in migratory neural-crest cells (Yu et al., 2005). Gli1-dependent signaling is needed to regulate CNC cells (Brewster et al., 1998; Ahlgren et al., 2002). Axin2 is a region-specific factor, and is required for CNC-dependent skeletogenesis, but it is not important for mesoderm-derived parietal-bone osteoblasts (Yu et al., 2005). Axin2 regulates the calvarial bone and development (McGee-Lawrence et al., 2013). However, during the CNC induction, BMP signaling is a vital growth factor that emerged earlier. BMP4 and BMP7 were both found expressed at the time of CNC induction, and the balance between Noggin and BMPs signals are also needed for the CNC delamination. BMP2 was also identified as a factor that required for the production of migratory cranial neural crest cells (Kanzler et al., 2000). We previously proposed a potential gene-regulatory network from the patterning of neural-crest cells to calvaria development (Wu et al., 2017), where we proposed that CNC cells may provide instructive cues for their derived cells or tissues, and these inherent cues may work to maintain the properties of neural crest-derived tissues. BMP signaling mediated CNC is probably needed to produce or maintain suture-derived stem cells. More researches or BMP based lineage tracing can be used to evaluate the detailed contribution of BMPs to calvarial stem cells at embryonic and adult stages.

### BMP Signaling in Adult Calvarial Stem-Cell Niche

**FIGURE 2:**
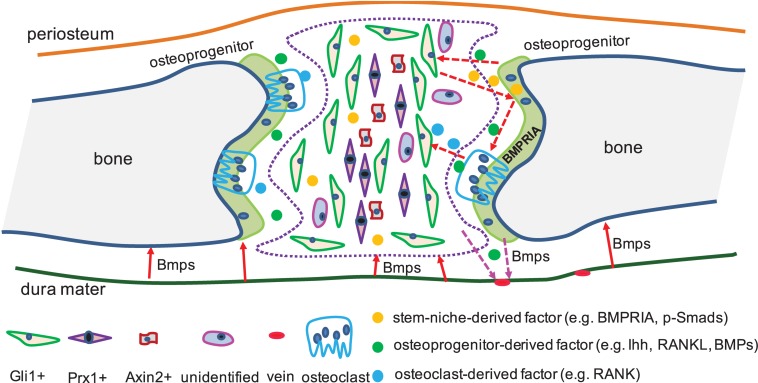
BMP signaling interacts with different factors in suture-derived stem cells. In cranial sutures, identified and unidentified suture-derived stem cells were exhibited (The number of the labels indicate the population of stem-niche). Dura mater was found expressed BMPs which were required for the calvarial bone development (solid arrow in red). CNC- and mesoderm-derived preosteoblasts expressed BMPs (e.g., BMP2/BMP4), which were needed for the proper shaping of cerebral veins (dotted arrow in pink). Osteoprogenitors released Ihh, which was required by the suture-derived stem cells (e.g., Gli1+), and this process can be mediated by BMP signaling (dotted arrow in red). Besides, BMP mediating Ihh signaling and RANKL can work together to regulate the activities of osteoclasts (dotted arrow in red).

Bone morphogenetic protein ligands, BMPRs, and intracellular Smads are expressed in suture mesenchyme cells (Opperman, 2000; Ishii et al., 2015). Noggin expression is highly related to patent sutures (Warren et al., 2003), and improper Noggin expression can prevent cranial suture fusion (Warren et al., 2003). BMPRIA mutations in osteoclasts, osteoblasts, or cartilage result in defective bone remodeling or growth (Mishina et al., 2004; Kamiya et al., 2008; Okamoto et al., 2011; Jing et al., 2013, 2015), and constitutively activated BMPRIA in neural-crest cells results in craniosynostosis (Komatsu et al., 2013). BMP2 is ectopically expressed in Gli3 mutant mesenchyme, which can lead to abnormal osteoblasts differentiation (Tanimoto et al., 2012). Osteoprogenitors from Gli1+ suture-derived stem cells was found to release Ihh, which is required to maintain the homeostasis of Gli1+ suture-derived stem cells, and this process is fine-tuned by BMPRIA (Guo et al., 2018). Further, the paracrine BMP2/BMP4, secreted by preosteoblasts, is principally required for the morphogenesis of dural cerebral veins (Tischfield et al., 2017), which then influence the state of the suture-derived stem niche. This suggested the unrecognized importance of tissue–tissue interactions in suture biology. Therefore, we proposed a BMP diagram where different factors that from osteoprogenitors, osteoclasts, and suture-derived stem cells can coordinate each other at spatial and temporal levels, in either a paracrine or an autocrine manner, to converge together to fine-tune the homeostasis of the suture-derived stem cells through precise communications (Figure 2).

## BMP Signaling in Calvarial Regeneration

### BMPs and Calvarial Regeneration

The superior ability of CNC-derived calvarial bone to regenerate prompted a new idea for craniofacial construction, called endogenous calvarial regeneration. Ideally, a suitable cytokine or drug would be sufficient to initiate the endogenous program for bone healing. FGF and Wnt signaling are able to quicken bone healing from injury (Xu et al., 2007; Quarto et al., 2009, 2010, 2018; Behr et al., 2010; Ichikawa et al., 2015; Li et al., 2015; Hu et al., 2017; Wu et al., 2017; Doro et al., 2019). BMPs, receptors, and intracellular Smads have robust expressions in the periosteum after a fracture, and their expressions are detectable in endothelial cells that associated with new bone formation (Yu et al., 2010). However, the deletion of BMP2 in osteoblasts and vascular endothelial cells prior to the fracture results in no significant influence on fracture healing (McBride-Gagyi et al., 2015), which suggested that focusing on BMPs with distinct expressions at the fracture sites is not work reliably to heal the fractures or BMPs might play a redundant role. Recent evidence has shown that BMP9 functions at the fracture site, at the same time, BMP9 can function as an inductive signal to maintain proliferative capacities of suture-derived stem cells in the long term to differentiate into osteogenic lineages *in vivo* and *in vitro* (Song et al., 2017). More interestingly, BMP9 regulates Nell-1 activity, which is an osteo-inductive growth factor, and mutant Nell-1 results in significant cranial abnormalities (Song et al., 2017; Chen et al., 2019b). In our early publication, we found that Nell-1 in neural-crest-derived frontal bone is significantly expressed compared to that in mesoderm-derived parietal bones (Hu et al., 2017). Given the information, it will be a new strategy or direction that combining BMPs screening at the injury site and an emphasis of BMPs to induce the mobilization of the suture-derived stem cells to the fracture sites, to be considered to promote the potential application of BMPs therapy in the clinic to heal the injury.

### BMP Signaling Mediated Calvarial Stem Cells to Regeneration

Cranial sutures provide guidance for the development and regeneration of calvaria bone (Maruyama, 2019). The identified suture-derived stem cells exhibit a greater potency in regenerative medicine in the clinic. A complete removal of the sagittal suture, and the defects can be completely restored in 6 weeks (Park et al., 2016). The progeny of Gli1+ suture-derived stem cells are detectable after 2 weeks at parietal injury sites, and their continuous increase contributes to new bone formation (Park et al., 2016). The ablation of Gli1+ cells leads to growth arrest, suture fusion, and severe osteoporosis (Zhao et al., 2015). The progeny of Prx1+ cells migrate to the injury site to make new bone (Wilk et al., 2017). The transplantation of Axin2+ cells to the injury site can significantly improve healing efficacy within 2–4 weeks (Maruyama et al., 2016; Table 3). Suture-derived stem cells are suitable therapeutic targets to heal damaged calvaria (Ransom et al., 2016). BMPs have been evaluated to be functional to enhance the capacities of suture-derived stem cell. BMP2 encoding suture-derived stem cells is able to improve the healing of critical size cranial bone defect (Vural et al., 2017). Interestedly, BMPRIA is reported to be a principal factor to regulate the homeostasis of Gli1+ suture-derived stem cells. Disruption of BMPRIA results in diminished cranial sutures and minimal healing ability (Guo et al., 2018). It suggested that suitable modulation of BMPs in suture-derived stem cells can facilitate bone regeneration following calvarial bone damage.

**TABLE 3:** Calvarial injury model and stem-niches used in cranial bone regeneration.

Suture cells	Injury model	Progeny expression	Bone regeneration	References
Gli1+ cells	Rectangular defect crossing the sagittal suture	Bmpr1a loss in Gli1+ cells	Disrupted osteoclastogenic activity, severely impaired	Guo et al., 2018
Gli1+ cells	Calvarial injury to bone (1 m mice)	Detectable in Gli1+ in 2 weeks	Strongly labeled Gli1+ cells in a month	Park et al., 2016
Suture transplantation	Suture injury	Detectable on the surfaces of the transplants in 1 week	Bone regeneration in 1 month	Park et al., 2016
Suture stem cells	2 mm^2^ defect in mice centered at the sagittal suture	Significant injury closure in 2 weeks	Complete recovery in 4 weeks	Park et al., 2016
Suture stem cells	2 × 5 mm removal of sagittal suture	Newly formed bone in 3 weeks	Complete recovery in 6 weeks	Park et al., 2016
Gli1+ cells	2 mm^2^ defects in parietal bone (1 mm to sagittal suture)	Gli1+ cells detectable in 2 weeks	∼50% healing of injury in 4 weeks	Park et al., 2016
Suture stem cells	2 mm^2^ defects in parietal bone (0.5 mm to sagittal suture)		∼80% healing of injury in 4 weeks	Park et al., 2016
Suture stem cells	4 mm^2^ in rabbit parietal bone or at the sagittal suture		Suture injuries healed in 1 month	Park et al., 2016
Gli1+ cells	Ablation in Gli1+ cells	Most sutures patent in 1 month	Growth arrest and compromised repair in 2 months	Zhao et al., 2015
Prx1+ cells	2 mm^2^ in mouse frontal and parietal bone	Detectable in 10 days	New bone formed in 4 weeks	Wilk et al., 2017
Axin2+ cells	1.4 mm^2^ in mouse parietal bone	∼46% residing cells at the injury site in 4 weeks	∼98% derivative cells	Maruyama et al., 2016

## Summary

The BMP signaling pathway has been widely demonstrated to be a critical requirement for organogenesis, involved in a broad spectrum of regulation of cell proliferation, differentiation, and survival in stem cell niches. Calvarial bones with dual-tissue lineages exhibit distinct osteogenic capacities and regeneration. The inherent cue of BMP signaling is highly associated with the regulation of calvarial osteoblasts *in vivo* and *in vitro*. More importantly, the modulation of BMP signaling is able to influence osteogenic potential, ossification and the homeostasis of suture-derived stem cells, which can significantly contribute to the repair of craniofacial bone defects. As further investigation of BMP signaling in suture stem-niches continues, a suitable BMP that itself is able to accelerate the regeneration, and its novel function in the mobilization of endogenous suture-derived stem cells will be a promising therapeutic target to be applied to heal bone defects in the clinic.

## Author Contributions

YY, HX, ZL, XZ, and MY: material collections and analysis. GC, ZL, and MW: design, review, and proofreading of the manuscript. All of the authors agreed with the submission of the current version of the manuscript.

## Conflict of Interest

The authors declare that the research was conducted in the absence of any commercial or financial relationships that could be construed as a potential conflict of interest.
